# Comparison of Water Pipes vs Other Modes of Cannabis Consumption and Subsequent Illicit Drug Use in a Longitudinal Cohort of Young Swiss Men

**DOI:** 10.1001/jamanetworkopen.2021.3220

**Published:** 2021-04-06

**Authors:** Dai-Hua Tsai, Simon Foster, Stéphanie Baggio, Gerhard Gmel, Meichun Mohler-Kuo

**Affiliations:** 1 Department of Child and Adolescent Psychiatry and Psychotherapy, University Hospital of Psychiatry Zurich, University of Zurich, Zurich, Switzerland; 2 Division of Prison Health, Geneva University Hospitals and University of Geneva, Thônex, Switzerland; 3 Office of Correction, Department of Justice and Home Affairs of the Canton of Zurich, Zurich, Switzerland; 4 Addiction Medicine, Lausanne University Hospital and University of Lausanne, Lausanne, Switzerland; 5 Addiction Switzerland, Lausanne, Switzerland; 6 Centre for Addiction and Mental Health, Toronto, Ontario, Canada; 7 Faculty of Health and Social Sciences, University of the West of England, Frenchay Campus, Bristol, United Kingdom; 8 La Source, School of Nursing Sciences, HES-SO University of Applied Sciences and Arts of Western Switzerland, Lausanne, Switzerland

## Abstract

**Importance:**

There are concerns that the use of water pipes to consume cannabis is associated with increased risks of engaging in more addictive behaviors.

**Objective:**

To examine whether consuming cannabis with a water pipe was associated with later consumption of other illicit drugs compared with not using a water pipe.

**Design, Setting, and Participants:**

The Cohort Study on Substance Use Risk Factors (C-SURF) was a population-based study, recruiting 5987 Swiss men aged 18 to 25 years from 3 of 6 Swiss Armed Forces recruitment centers (response rate: 79.2%). The baseline assessment (t0) was done from 2010 to 2012, first follow-up (t1) from 2012 to 2014, and second follow-up (t2) from 2016 to 2018. Our sample included men who participated in both t0 and t2 assessments and used cannabis but no other illicit drugs at t0. Data analysis was performed from July 2020 to January 2021.

**Exposures:**

Cannabis use frequency and route of administration from self-administered questionnaires completed at t0 and t2.

**Main Outcomes and Measures:**

Outcome measures were initiation of illicit drug use and cannabis use disorder, identified by the Cannabis Use Disorder Identification Test. To examine whether water pipe use at t0 was associated with illicit drug use at t2, multivariable logistic regression analysis was performed.

**Results:**

Among 1108 Swiss male cannabis users who did not use other illicit drugs at t0, the mean (SD) age was 20 (1.2) years, 617 (55.7%) were from Switzerland’s French-speaking region, and 343 (30%) used water pipes to consume cannabis. Water pipe users at t0 were more likely to use other illicit drugs at t2 compared with water pipe nonusers (adjusted odds ratio [aOR], 1.54; 95% CI, 1.10-2.16). The odds of using middle-stage drugs (including stimulants, hallucinogens, and inhaled drugs) at t2 were increased for water pipe users (aOR, 1.61; 95% CI, 1.13-2.29). Water pipe use at t0 was not associated with cannabis use disorder at t2 after adjusting for cannabis use frequency.

**Conclusions and Relevance:**

This cohort study’s results suggest that, among Swiss young men, water pipe use is associated with other illicit drug use later in life, particularly middle-stage illicit drugs. Preventive programs must focus on the potential of later harm to cannabis users who use water pipes but have not yet started taking other illicit drugs.

## Key Points

**Question:**

Is there an association between consuming cannabis with a water pipe and later consumption of other illicit drugs?

**Findings:**

In this longitudinal cohort study of 1108 men, both unadjusted and adjusted models showed that consumption of cannabis with a water pipe at baseline was associated with the use of other illicit drugs at follow-up compared with not using a water pipe.

**Meaning:**

Policy campaigns designed to reduce the prevalence and risks associated with cannabis use should not only target cannabis frequency, but also the water pipe route of administration.

## Teaser

This cohort study investigates whether consumption of cannabis with a water pipe is associated with eventually using illicit drugs compared with cannabis consumption without water pipes.

## Introduction

Cannabis is a commonly used illicit drug worldwide, especially among young people.^1^ The highest prevalence has been reported among those aged 15 to 24 years.^2,3^ The current wave of legalization and commercialization of cannabis for recreational and medical purposes may popularize the perception that cannabis is harmless.^4^ Adverse health outcomes associated with cannabis use include the risk of addiction, declines in cognitive and motor function, and associated risks such as motor vehicle crashes, impaired brain function, mental illness, impaired lung function, and other health outcomes.^5,6,7,8^ The burden of disease associated with cannabis use is substantial.^9^ Therefore, understanding cannabis use is crucial for both the prevention and treatment of a variety of associated disorders.

Various routes of cannabis administration exist, including inhalation via smoking and vaporization and ingestion of edible products.^10^ Among them, smoking appears to be the most common mode of consumption, followed by water pipes, bongs, or hookahs.^11,12,13^ Some cannabis industry media claim that using water pipes is cleaner and safer than smoking it, although medical and public health sources have raised concerns about residual solvents and pesticides and associated negative health outcomes.^14^

Cross-sectional analyses have shown that water pipe users report a higher frequency of cannabis use and more illicit drug use than non–water pipe cannabis users.^12^ The use of a water pipe hastens its penetration into bodily tissues.^15^ Its ingredients flood rapidly into the body and produce more intense psychotropic effects, leading to the consumption of larger amounts and increasing the risk of addiction.^16^ Using water pipes to consume cannabis generates a rapid and intense effect comparable to the effects of other illicit drugs.^13,17,18,19^ Increasing the frequency of cannabis use also can result in individuals electing to consume more illicit drugs.^20^

Water pipe is the second most common method of cannabis use among young people aged 14 to 23 years.^12,13,19^ Although certain studies have detected an association between cannabis use and the subsequent use of other illicit drugs, the progression from cannabis use to other illicit drugs remains unknown.^21^ In particular, the aforementioned studies did not investigate the association between route of administration of cannabis and the later illicit drug use. Our study aimed to examine whether water pipe–based cannabis consumption at the age of 20 years would be associated with the initiation of other illicit drugs by age 25 years. For this, we analyzed data from a population-based sample of young Swiss men who were surveyed twice for cannabis and other illicit drug use, with an interval of 5.5 years between the 2 assessments.

Other illicit drug use is often categorized as a binary outcome to indicate whether a person uses them or not. To distinguish the stage of involvement with different drugs,^22,23^ we added 2 additional outcome categories: middle-stage and final-stage illicit drug use. The middle stage included 9 substances (including stimulants, hallucinogens, and inhaled drugs), and the final stage included 6 substances (heroin, ketamine, GHB/GBL [gamma hydroxybutyrate/gamma-butyrolactone], research chemicals, crystal meth, and spice). Distinguishing these 2 kinds of drug use helps to consider different progression of illicit drug use.^24^

## Methods

### Participants and Study Design

Data were drawn from 2 waves of assessments of the Cohort Study on Substance Use Risk Factors (C-SURF) in young Swiss men, a representative study of a noninstitutionalized sample. Detailed study information, including study process, consent, and questionnaires, can be found on the C-SURF website.^25^ The methods have been described elsewhere in detail.^26,27,28^ Participants were recruited at 3 of 6 Swiss Armed Forces centers recruiting men for military service, representing 21 of 26 Swiss cantons. All Swiss men must go through this recruitment process to determine their eligibility between the ages of 18 and 24 years. Among the 7563 men who provided informed consent, 5987 (79.2%) completed a written questionnaire between 2010 and 2012 (t0, mean [SD] age: 20 [1.2] years). The second follow-up assessment (t2, mean [SD] age: 25.5 [1.3] years) was conducted between 2016 and 2018.

We examined cannabis users who claimed not to have used any other illicit drugs at baseline. Only 1135 participants reported using cannabis but not any other illicit drug at t0. Among them, 27 did not indicate the route of administration. Their missing values were excluded listwise, resulting in a final sample of 1108 participants.

The C-SURF study protocol was approved by the Human Research Ethics Committee of the Canton of Vaud, and written informed consent was obtained from all participants. The reporting of this study followed the Strengthening the Reporting of Observational Studies in Epidemiology (STROBE) reporting guideline.^29^

### Measures

#### Other Illicit Drug Use

The use of illicit drugs other than cannabis during the previous 12 months was determined by asking participants whether they had either used or not used the following 15 categories: (1) hallucinogens, magic mushrooms, psilocybin, peyote, and mescaline; (2) other hallucinogens (lysergic acid diethylamide, phencyclidine/angel dust, 2,5-dimethoxy-4-bromophenethylamine [2-CB], and 2,5-dimethoxy-4-iodophenethylamine [2-CI]); (3) *Salvia divinorum*; (4) speed; (5) amphetamines, methamphetamines, and amphetaminsulfate (eg, dextroamphetamine or benzedrine); (6) cocaine, crack, and freebase; (7) poppers (amyl nitrite and butyl nitrite); (8) solvents for sniffing; (9) 3,4-methylenedioxy-methylamphetamine (MDMA or ecstasy); (10) crystal meth (ice); (11) heroin; (12) ketamine (special K), dextromethorphan (DXM); (13) GHB/GBL and 1,4-butanediol (1,4-BD); (14) research chemicals (eg, mephedrone, butylone, and methedrone); and (15) spice or similar substances (synthetic cannabis). A summed score of substances (ranging from 0 to 15) was calculated at both t0 and t2. We then dichotomized that summed score into the no illicit drug use category and the illicit drug use category.

To consider distinct stages of progression among different illicit drugs, we further categorized users into middle-stage and final-stage drug users by their choice of drugs, according to a previous study.^24^ Middle-stage drugs included 9 substances (see numbers 1-9 from the aforementioned list), and the final-stage drugs included 6 substances (see numbers 10-15 from the aforementioned list).

#### Cannabis Use

If participants responded yes to whether they had used cannabis during the previous 12 months (whether at t0 or t2), we asked them to estimate the frequency of their use (not at all, up to once monthly, 2-4 times per month, 2-3 times per week, 4-5 times per week, and every day or almost every day). We also asked about their main route of cannabis administration, with response options including cannabis cigarettes (joints), water pipes (bong), and mixed with food. We categorized participants into 2 groups: water pipe users vs water pipe nonusers (ie, using joints or consuming cannabis in food).

#### Cannabis Use Disorder

Cannabis use disorder (CUD) was assessed using the Cannabis Use Disorder Identification Test (CUDIT). The cutoff for CUD (binary variable: CUD vs no CUD) was set at 8 points on a scale from 0 to 40.^30^

#### Covariates

Data on respondents’ sociodemographic characteristics were collected at baseline, including their age (in years), linguistic region (French or German speaking), highest achieved education level (obligatory school, secondary vocational school, or tertiary school), and level of financial autonomy (independent, partially independent, or fully dependent). We created a dichotomized variable, called region, to indicate whether participants lived in a rural or urban setting, defining rural as communities with fewer than 10 000 inhabitants. Data also were collected on whether respondents perceived any peer pressure to use cannabis (yes or no). We asked whether they had ever used or consumed any tobacco products, defining such use in terms of satisfying at least 1 of 7 scenarios: smoked 50 or more cigarettes; used a bong 10 or more times; used snus 10 or more times; used snuff 10 of more times; chewed tobacco 10 or more times; smoked 25 or more cigars or cigarillos; or smoked 25 or more pipes.

To address the possibility that water pipe and illicit drug users were simply at higher risk for substance use in general, we asked questions to assess each respondent’s level of sensation seeking. For this purpose, respondents were asked to rate their level of agreement with each of 8 sensation-seeking statements, such as “I like wild parties” or “I would like to try bungee jumping” (Cronbach α = 0.81), with each question answered using a 5-point rating scale. The sensation-seeking scale is 1 to 5, with highest score (5) indicating a strong inclination to engage in risky activities. These 8 scores then were averaged to generate a mean sensation-seeking score.

To address the possibility that the findings were skewed by a subset of young adults with behavioral or psychiatric disorders, antisocial personality disorder (ASPD) was controlled for as well, evaluated using the Mini International Neuropsychiatric Interview.^31^ Responses were dichotomized to indicate the absence or presence of each symptom. ASPD was defined as the presence of at least 2 symptoms before the age of 15 years and 3 symptoms after age 15 years.

### Statistical Analysis

First, Pearson χ^2^ analysis and independent-sample *t* tests were performed to compare cannabis users who used vs did not use a water pipe at t0. There were 3 main outcomes of interest: any illicit drug use (yes or no); middle-stage drug use (yes or no); and final-stage drug use (yes or no). For this analysis, the dependent variable was the proportion using any 1 of the 15 categories of illicit drugs listed previously when reassessed at t2. Logistic regression analysis was then performed with illicit drug use at t2 set as the dependent variable, and water pipe use at t0 as the covariate. Odds ratios (ORs) were calculated, adjusting for (1) frequency of cannabis use; (2) sociodemographic characteristics, including age, linguistic region, highest achieved education level, level of financial autonomy, and urban or rural region; and (3) other factors, including self-perceived peer pressure to use cannabis (yes or no), whether any tobacco products had ever been tried (yes or no), ASPD (yes or no), and mean sensation-seeking score. These analyses were performed: (1) unadjusted, (2) adjusted for cannabis frequency alone, and (3) adjusted for cannabis frequency plus all the other aforementioned covariates.

All statistical analyses were 2-tailed, with *P* ≤ .05 set as the criterion for statistical significance. Statistical analyses were conducted using Stata Special Edition statistical software version 15.0 (StataCorp) from July 2020 to January 2021.

## Results

Among the 1108 Swiss male cannabis users who did not use other illicit drugs at the baseline assessment (t0), the mean (SD) age was 20.0 (1.2) years, 617 (55.7%) were from Switzerland’s French-speaking region, and 343 (30%) used water pipes to consume cannabis. Compared with the reference group (3588 participants), cannabis users who reported no illicit drug use at t0 were more likely to report having achieved a tertiary-level education (291 [26.5%] vs 788 [22.2%]; χ^2^ = 20.8; *P* < .001), being more financially dependent (429 [38.9%] vs 1214 [34.1%]; χ^2^ = 20.3; *P* < .001), rating higher levels of sensation seeking (mean [SD] score, 3.3 [0.02] vs 2.9 [0.01]; *P* < .001), meeting the criteria for ASPD (249 participants [22.7%] vs 395 participants [11.2%]; χ^2^ = 92.1; *P* < .001), and reporting peer pressure to smoke cannabis (411 [37.5%] vs 240 [6.8%]; χ^2^ = 647.0; *P* < .001). Among cannabis users who did not use illicit drugs at t0, 966 (87.2%) reported having used some tobacco product. However, they were no different in mean age or linguistic region compared with cannabis nonusers (Table 1).

**Table 1.** Sample Characteristics Comparing Cannabis Nonusers and Cannabis Users With No Reported Illicit Drug Use at Baseline

Baseline variables	Participants, No. (%)		χ^2^ Value	*P* value
	Cannabis nonusers (reference group) (n = 3588)	Cannabis users without illicit drug use (n = 1108)		
Age, y				
<20	1810 (50.5)	549 (49.5)	0.3	.60
≥20	1778 (49.5)	559 (50.5)		
Linguistic region				
German	1599 (44.6)	491 (44.3)	0.0	.88
French	1989 (55.4)	617 (55.7)		
Education				
Obligatory school	1726 (48.6)	561 (51.0)	20.8	<.001
Secondary or vocational school	1035 (29.2)	248 (22.6)		
Tertiary school	788 (22.2)	291 (26.5)		
Region				
Rural	2198 (61.4)	644 (58.1)	3.7	.054
Urban	1384 (38.6)	464 (41.9)		
Financial autonomy				
Independent	850 (23.9)	195 (17.7)	20.3	<.001
Partially independent	1495 (42.0)	479 (43.4)		
Dependent	1214 (34.1)	429 (38.9)		
Antisocial personality disorder				
No	3129 (88.8)	848 (77.3)	92.1	<.001
Yes	395 (11.2)	249 (22.7)		
Peer pressure^a^				
No	3270 (93.2)	685 (62.5)	647.0	<.001
Yes	240 (6.8)	411 (37.5)		
Ever used any tobacco^b^				
No	1967 (54.8)	142 (12.9)	603.8	<.001
Yes	1621 (45.2)	966 (87.2)		
Sensation-seeking score, mean (SD)^c^	2.9 (0.01)	3.3 (0.02)	−15.0^d^	<.001

^a^ Refers to peer pressure to smoke cannabis.

^b^ The ever used any tobacco category includes smoking at least 50 cigarettes, using a bong, snus, or snuff at least 10 times, chewing tobacco at least 10 times, and smoking at least 25 cigars, 25 cigarillos, or 25 pipes.

^c^ The sensation-seeking score scale is 1 to 5, with higher scores indicating greater likelihood of engaging in risky activities.

^d^ This is a *t* value, not χ^2^.

Table 2 summarizes the comparison of cannabis users and illicit drug nonusers who reported using vs not using a water pipe at baseline, in terms of the proportion who reported having initiated any illicit drug use besides cannabis, middle-stage drug use, and final-stage drug use at t2. Among the 343 cannabis users who used water pipes at baseline, 241 (70.3%) were still not using other illicit drugs, 102 (29.7%) were using any illicit drugs, 89 (25.9%) were considered to be using middle-stage drugs, and 13 (3.8%) were using final-stage drugs. Among the 765 cannabis users who did not use a water pipe at t0, 631 (82.5%) were not using other illicit drugs at t2. Among cannabis users who reported illicit drug use at t2, a significantly greater proportion of water pipe users reported any illicit drug use (any illicit drug use at t2: 29.7% vs 17.5%; *P* < .001; χ^2^ = 21.1) or middle-stage drug use (25.9% vs 14.9%; *P* < .001; χ^2^ = 19.3) compared with those who did not use water pipes. No such association existed between baseline water pipe use and final-stage illicit drug use at t2.

**Table 2.** Comparison of Level of Illicit Drug Use at Final Follow-up in Cannabis Users Using vs Not Using a Water Pipe at Baseline

Level of illicit drug use	Baseline water pipe use, participants, No. (%)		χ^2^ Value	*P* value
	Yes (n = 343)	No (n = 765)		
Using an illicit drug at final follow-up				
None	241 (70.3)	631 (82.5)	21.1	<.001
Any	102 (29.7)	134 (17.5)		
Using middle-stage drug at final follow-up^a^				
None	254 (74.1)	651 (85.1)	19.3	<.001
Any	89 (25.9)	114 (14.9)		
Using final-stage drug at final follow-up^b^				
None	330 (96.2)	745 (97.4)	1.13	.29
Any	13 (3.8)	20 (2.6)		

^a^ Middle-stage drugs include hallucinogens, magic mushrooms, psilocybin, peyote, and mescaline; other hallucinogens (lysergic acid diethylamide, phencyclidine or angel dust, 2C-B, and 2C-I); *Salvia divinorum*; speed; amphetamine, methamphetamine, and amphetaminsulfate (eg, dextroamphetamine or benzedrine); poppers (amyl nitrite and butyl nitrite); solvent sniffing; ecstasy and MDMA; and cocaine, crack, and freebase.

^b^ Final-stage drugs include crystal meth (ice); heroin; ketamine (special K), DXM; GHB/GBL and 1,4-BD; research chemicals (eg, mephedrone, butylone, and methedrone); spice or similar substances (synthetic cannabis).

Table 3 summarizes the results of 3 logistic regression models for each of the 3 outcomes of interest: any illicit drug use, middle-stage drug use, and final-stage drug use at t2. Of the 3 models per outcome, model A is unadjusted, model B is adjusted only for cannabis use frequency, and model C is adjusted for cannabis use frequency and all the other covariates of interest. All 3 models demonstrated a significant association between baseline water pipe use and both any illicit drug use and middle-stage drug use at final follow-up. Water pipe users at t0 were more likely to use other illicit drugs at t2 compared with water pipe nonusers (adjusted odds ratio [aOR], 1.54; 95% CI, 1.10-2.16). The odds of using middle-stage drugs (including stimulants, hallucinogens, inhaled drugs) at t2 were increased for water pipe users (aOR, 1.61; 95% CI, 1.13-2.29). This fully adjusted odds ratio of 1.61 is equivalent to a Cohen *d* of 0.2, indicative of a small effect.^32^ None of the 3 models (A, B, or C) revealed any significant association between baseline water pipe use and final-stage illicit drug use. The aOR for final-stage drug use in our fully adjusted regression model (into which all potential covariates were entered) was only 1.02 (95% CI, 0.46-2.27).

**Table 3.** Results of Logistic Regression on the Association of Baseline Water Pipe Use With Illicit Drug Use at Final Follow-up

Illicit drug use outcome	OR (95% CI)		
	Model A^a^	Model B^b^	Model C^c^
Any	1.99 (1.48-2.68)	1.60 (1.17-2.20)	1.54 (1.10-2.16)
Middle-stage drugs^d^	2.00 (1.46-2.74)	1.62 (1.16-2.27)	1.61 (1.13-2.29)
Final-stage drugs^e^	1.47 (0.72-2.98)	1.21 (0.57-2.59)	1.02 (0.46-2.27)

Abbreviation: OR, odds ratio.

^a^ Model A is unadjusted.

^b^ Model B is adjusted for cannabis use frequency.

^c^ Model C is adjusted for cannabis use frequency, age, linguistic region, highest achieved education level, level of financial autonomy, urban or rural residence, perceived peer pressure to use cannabis, past use of tobacco product, antisocial personality disorder, and sensation-seeking score.

^d^ Middle-stage drugs include hallucinogens, magic mushrooms, psilocybin, peyote, and mescaline; other hallucinogens (lysergic acid diethylamide, phencyclidine or angel dust, 2C-B, and 2C-I); *Salvia divinorum*; speed; amphetamine, methamphetamine, and amphetaminsulfate (eg, dextroamphetamine or benzedrine); poppers (amyl nitrite and butyl nitrite); solvent sniffing; ecstasy and MDMA; and cocaine, crack, and freebase.

^e^ Final-stage drugs include crystal meth (ice); heroin; ketamine (special K), DXM; GHB/GBL and 1,4-BD; research chemicals (eg, mephedrone, butylone, and methedrone); spice or similar substances (synthetic cannabis).

Table 4 summarizes the results of 3 binary logistic regression models: the first is an unadjusted model assessing the association of baseline water pipe use with the rate of ultimate (t2) CUD; the second is an unadjusted model evaluating the association of baseline cannabis use frequency with ultimate CUD; and the third combines the 2 potential associations of CUD. Separately, both the baseline use of water pipes (water pipe use: OR, 1.57 [95% CI, 1.07-2.33]) and baseline frequency of cannabis use (eg, 2-4 times/mo: OR, 3.51 [95% CI, 2.22-5.53]; daily use: OR, 14.69 [95% CI, 5.59-38.59]) were associated with significantly increased odds of CUD at t2. However, when combining the 2 covariates into a single model, only baseline cannabis use frequency remained associated with CUD at final follow-up (eg, 2-4 times/mo: OR, 3.41 [95% CI, 2.14-5.43]; daily use: OR, 12.89 [95% CI, 4.76-34.89]).

**Table 4.** Association of Baseline Water Pipe Use and Cannabis Frequency With the Odds of Cannabis Use Disorder at Final Follow-up

Variable	OR (95% CI)		
	Model 1^a^	Model 2^b^	Model 3^c^
Baseline water pipe use			
No	1 [Reference]	NA	1 [Reference]
Yes	1.57 (1.07-2.33)	NA	1.08 (0.71-1.65)
Baseline cannabis frequency			
0-1 times/mo	NA	1 [Reference]	1 [Reference]
2-4 times/mo	NA	3.51 (2.22-5.53)	3.41 (2.14-5.43)
2-3 times/wk	NA	5.10 (2.74-9.47)	4.95 (2.60-9.40)
4-5 times/wk	NA	9.04 (3.59-22.79)	8.88 (3.50-22.50)
Daily	NA	14.69 (5.59-38.59)	12.89 (4.76-34.89)

Abbreviations: NA, not applicable; OR, odds ratio.

^a^ Model 1 is unadjusted.

^b^ Model 2 is unadjusted.

^c^ Model 3 is adjusted for baseline cannabis use frequency.

## Discussion

In this cohort study of young Swiss men, we found that water pipe–based cannabis use was associated with other illicit drug use 5.5 years later. Moreover, the association persisted even after adjusting for a wide array of potentially confounding factors, such as cannabis use frequency; user age, linguistic region, education level, residential setting (rural vs urban), and level of financial autonomy; past tobacco product use; the presence of peer pressure to use cannabis; and personality attributes such as sensation-seeking and ASPD.

Our findings contribute to previously published evidence suggesting that water pipe use might place users at increased risk for initiating the use of other illicit drugs among those who do not already do so. An earlier cross-sectional analysis of the C-SURF population revealed that water pipe users had a higher prevalence of illicit drug use.^12^ The authors of that article speculated that using a water pipe to use cannabis might be comparable to using harder drugs, because of the rapid, hyperintense effect that results from such hastened absorption.

Our analysis is consistent with the hypothesis that water pipe–based cannabis use is a problematic mode of administration and is associated with illicit drug use, above and beyond cannabis use frequency.^12^ Our results are comparable to those of cross-sectional studies^12,33^ that have identified associations between water pipe use and CUD. Our data also suggest that cannabis use frequency might play an important role in the association between water pipe use and CUD. Many water pipe cannabis users, although not illicit drug users at baseline, initiated the use of other illicit drugs, even after the analysis was adjusted for cannabis use frequency. Our study supports the hypothesis that water pipe use may have a long-term association. One possible mechanism could be associated with the increased intensity of psychotropic stimulus that water pipe users often seek, in steadily greater amounts, thereby placing them at higher risk for addiction to cannabis and leading them to seek other potentially more psychotropic drugs to either maintain or enhance the desired results.^16^

In a study^34^ that used a modeling procedure to describe initiation sequences, the most likely model started with cannabis before progressing to other illicit drugs. In a study^35^ of Native American adolescents, substantial progression from cannabis use to the use of harder substances was identified. In both studies, a sizeable proportion of cannabis users went on to use other illicit drugs.^21^ Our study extends these previous findings pertaining to the route of cannabis administration by specifically identifying the association of water pipe use and the use of middle-stage rather than final-stage drugs. For this, the fully adjusted odds ratio was 1.61, equivalent to a Cohen *d* of 0.2, indicative of a small effect.^32^

We investigated 2 different kinds of illicit drug use: middle-stage drugs (eg, hallucinogen, speed, and poppers) and more problematic final-stage drugs (eg, heroin, ketamine, and crystal meth). Of these 2 drug categories, baseline water pipe use was significantly associated with middle-stage drugs only. There are a variety of potential reasons for this. First, because there were far fewer final-stage than middle-stage drug users at the time of our final follow-up evaluation, it might be that the study merely lacked the statistical power to detect any association. Arguing against this, however, is that the aOR for final-stage drug use in our fully adjusted regression model (into which all potential covariates were entered) was only 1.02. A second reason could be that the duration of follow-up was too short. It might be, for example, that the progression from cannabis to final-stage drug use takes longer than 5 years; in essence, many users must pass through the phase of using middle-stage drugs before progressing to final-stage drugs. As such, were we to perform a further evaluation of the same sample, perhaps 10 years from baseline, both the number of final-stage drug users might increase and an association between water pipe and final-stage drug use might become more apparent.

### Limitations

There are some limitations in this study. First, our study did not analyze the use of vaping, because it was asked about at the follow-up assessment only. Second, we were unable to determine at which time point participants started to consume illicit drugs, which could be anytime during the 5.5 years. As such, other factors might have played an even bigger role in the progression from cannabis to other drugs. Further investigations with a longitudinal design will be required to overcome this limitation. Third, because all data were self-reported, data accuracy cannot be definitively determined. Fourth, our sample was restricted to men who all were approximately aged 20 years at the time of first assessment. Consequently, extrapolating our findings to men of different ages and to women needs to be done with great caution. Further studies will need to examine whether these findings will be the same in the female population.

## Conclusions

Our data suggest that policy campaigns designed to reduce the prevalence and risks associated with cannabis use should target not only cannabis frequency but also the water pipe route of administration, which appears to be associated with greater risk of progression to other illicit drugs than other modes of drug administration among young men. Although further research to confirm our findings in other populations and during longer periods remain necessary, it seems reasonable to advise the public about this potential health risk to water pipe users.
